# The boiling frog syndrome: A radiologist’s perspective

**DOI:** 10.2349/biij.6.4.e36

**Published:** 2010-10-01

**Authors:** N Mohd. Ramli, Y Faridah

**Affiliations:** Department of Biomedical Imaging, Faculty of Medicine, University of Malaya, Kuala Lumpur, Malaysia

Recently, an interesting entry has been posted on Facebook about the boiling frog syndrome. This syndrome is a metaphoric account of a frog being placed in a tub of water that is slowly heated. However, the frog will not notice the temperature difference until it is slowly boiled to death.

The description is merely anecdotal; however, this has not stopped several scientists from trying to prove the theory right, which makes for more interesting reading [1]. What is even more interesting is that this syndrome has been linked to our apathy to climate change, the economic meltdown, regime changes and career-related stress [2]. At the basic level, the boiling frog syndrome is a cautionary warning against complacency.

Apart from the more traditional role of interpreting images, the radiologist’s role within the health care system has expanded to encompass economic gatekeeping, political advocacy, public health delivery, patient safety, quality of care and information management [3]. However, the radiologist’s unique position lends itself to exposure to this syndrome. While trying to shoulder the many demands and responsibilities demanded of the profession – in other words, the metaphorical rise of temperature – radiologists who are unable to cope may just sit there and accept the situation, however far from ideal it may be.

It is not impossible to take charge and get back to the ideal situation. One of the most stressful roles for a radiologist is being the gatekeeper of diagnostic imaging. A gatekeeper can be defined as someone who is positioned between an organisation and the individuals who wish to utilise the resources within that organisation [4]. The radiologist is not the primary caregiver and is usually at a disadvantage when discussing imaging options or negotiating urgency when clinicians demand it.

One of the approaches that radiologists can use to assist appropriate diagnostic imaging utilisation is clinician education [5, 6]. This may be done by conducting one-to-one discussions, regular clinico-radiological conferences or roadshows to talk about appropriate diagnostic examinations. These methods foster greater mutual understanding and may hopefully lead to the development of diagnostic guidelines for certain diseases.

However, the communication methods above are fairly time-consuming and may not work well in a busy and high-output hospital. Some have advocated harnessing the advances in imaging and information technology to aid in this endeavour. Giving clinicians easy access to view available imaging examinations and reports may stop unnecessary repetition of examinations [7]. Further steps could be added to a computerised order system to display a list of appropriate imaging modalities for certain diseases, to highlight a recent similar examination that had been performed or to advise on certain contra-indications for specific investigations. Other useful applications include assigning keywords in diagnosis. These steps may assist the clinicians in making the right choice while decreasing the amount of time arguing with radiologists about inappropriate investigations [2]. The American College of Radiology (ACR)’s appropriateness criteria or the Royal College of Radiologist UK’s ‘Making the best use of clinical radiology services’ could be used as a template.

Fortunately, it is not all bad news for radiologists suffering from the ‘boiling frog’ syndrome. Continuous quality improvement (CQI), with the mantra of ‘start small, start early and keep working on it’ [8], has the ability to combat the ‘boiling frog’ syndrome by advocating for small continuous changes. CQI is defined as structured organisational processes for involving personnel in planning and executing a continuous flow of improvements to provide quality health care that exceeds expectations [8]. The key features of CQI are customer-mindedness, data collection, experimentation, and teamwork. Like the systems approach to error management, CQI methods attempt to anticipate problems rather than react to them. The difference is that in the former, these small changes encourage improvements in the organisation’s activities and outputs, while the latter relates to small changes as a result of complacency.

## SUMMARY

The boiling frog syndrome is a metaphor that can be applied to radiologists who remain complacent and unmoving in the face of rapid developments in their profession. Unfortunately, there is no magic potion to turn the frog into a prince. Stressful situations can, and will, occur in everyday practice. It is sometimes easier to back down and accept the situation, rather then taking a hard look and getting to the root of the problem in order to find a long and hopefully workable solution. The anecdotal syndrome serves as a reminder that complacency may add to the burden of the job and is best avoided.
